# Different Treatments on the Physicochemical Properties and Volatile Components of Tea Wine During Storage Period

**DOI:** 10.3390/molecules29245946

**Published:** 2024-12-17

**Authors:** Fang Huang, Yu-Hong Yan, Qing-Bo Yao, Meng-Na Li, Jun-Wei Ma, Zhi-Hong Zhang, Yan-Yan Huang, Xiang-Ze Jia

**Affiliations:** 1 Guangdong Provincial Key Laboratory of Intelligent Food Manufacturing, College of Food Science and Engineering, Foshan University, Foshan 528225, China; 16602749429@163.com (F.H.); 15220379281@139.com (Y.-H.Y.); 17543729909@163.com (Q.-B.Y.); mengnlimo@126.com (M.-N.L.); 2 School of Food Science and Engineering, South China University of Technology, Guangzhou 510640, China; femjw458@mail.scut.edu.cn; 3 School of Food & Biological Engineering, Jiangsu University, Zhenjiang 212013, China; zhihong1942@ujs.edu.cn

**Keywords:** tea wine, storage period, low-temperature plasma, volatile components

## Abstract

Tea wine has garnered significant attention due to its unique fusion of tea and wine flavors, as well as its alleged health benefits. This study aimed to investigate the effects of various treatments on the physicochemical properties of tea wine, including viable cell counts, pH, acidity, total ester content, tea polyphenol content, and volatile flavoring substances during the storage period. The findings indicated that tea wine subjected to low-temperature plasma (LTPS) treatment exhibited superior quality maintenance and an enhanced tea polyphenol content compared to untreated, UV-treated, and HTHP-treated tea wine. Analysis of volatile compounds revealed that the LTPS group exhibited the highest relative content of alcohols and esters (89.72%) during storage, thereby enhancing the fruity and sweet aroma of the tea wine. This study provides a theoretical basis for the application of low-temperature plasma technology in the storage of tea wine.

## 1. Introduction

Green tea is one of the most widely produced teas in China and has a long history of consumption. It accounts for approximately 70% of the total tea production and is the most consumed tea among consumers [1]. It is therefore evident that the enhancement of green tea value represents a crucial area of research. One potential avenue for achieving this objective is through the fusion of green tea with wine. This approach not only preserves the functionality of the tea but also introduces a novel variety and flavor to the wine [2]. Tea wine is a low-alcohol beverage produced through the fermentation of a mixture of tea leaves, glutinous rice, and microbiological agents, combining the refreshing flavor of tea with the smooth taste of wine [3]. Research has shown that tea wine possesses several beneficial properties, including the ability to alleviate fatigue, act as an antioxidant, regulate blood sugar levels, protect the nervous system, relieve gastrointestinal discomfort and enhance immune function [2,4,5]. Consequently, tea wine has gained popularity among consumers for its unique flavor and health benefits. The quality of tea wine remains a topic of significant interest among researchers.

Low-temperature plasma (LTPS) technology represents an innovative alternative to non-thermal food processing, offering numerous advantages such as enhanced food quality, operational efficiency, reduced energy consumption, and improved safety [6]. The generation of plasma is accompanied by several processes, including photoemission, cavitation, and the production of free radicals. These processes can directly influence the generation, degradation, and modification of a wide range of organic compounds [7]. In recent years, the application of this technology has expanded to various winemaking processes, including extraction, fermentation, aging, and sterilization [8]. It has been demonstrated that applying cold plasma to wine effectively reduces harmful biogenic amine levels while increasing catechin compound concentrations during storage [9]. Furthermore, plasma treatment of white wines resulted in enhanced phenolic concentrations and improved oxidative stability, thereby elevating the overall quality of the wines [10]. Additionally, atmospheric pressure cold plasma treatment increased anthocyanin content and color in Tempranillo red wine [11]. In conclusion, the utilization of plasma technology in tea wine treatment holds the potential to enhance both the organoleptic quality and nutritional value of the final product, while simultaneously mitigating the production of harmful substances. Nevertheless, a review of the literature reveals a scarcity of reports elucidating the mechanism of action of LTPS on food products. The existing literature on the impact of LTPS on the quality and aroma of alcoholic beverages during storage is limited, highlighting the need for further research in this area.

Therefore, this study was conducted to investigate changes in the physicochemical properties of tea wine during a storage period of 0–84 days using LTPS treatment, with the aim of comparing the effects of this treatment with those of three other treatments (UV treatment, high-temperature and high-pressure treatment, and no treatment). The physicochemical properties investigated included viable cell count, pH, acidity, total ester content, tea polyphenol content, alcohol content, color parameters, and changes in aroma components. The present study provides a theoretical foundation for the utilization of LTPS technology in the context of tea wine storage.

## 2. Results and Discussion

### 2.1. Effects on pH and Titratable Acidity of Tea Wine

**Figure 1:**
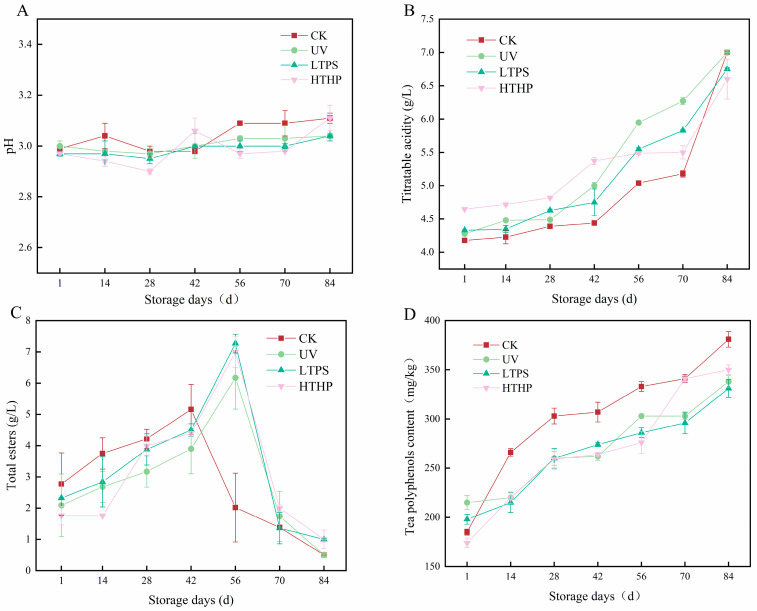
Changes in physicochemical indicators of tea wine during storage: pH (**A**), titratable acidity (**B**), total esters (**C**), tea polyphenols content (**D**). HTHP refers to high-temperature and high-pressure treatment at 121 °C for 20 min, UV refers to ultraviolet treatment for 20 min, LTPS refers to low-temperature plasma treatment at a voltage of 160 kV and a frequency of 70 Hz for 5 min, and CK refers to an untreated control.

The pH and titratable acidity (TA) of tea wine are key indicators of quality. For normally fermented products, the pH typically remains below 4, while the TA exceeds 3 g/kg. A low pH combined with high TA not only enhances the quality and flavor of fermented tea wine but also inhibits the growth of harmful microorganisms [12]. As shown in Figure 1A,B, the pH of the tea wines treated in various ways consistently remained around 3.0 over the course of the storage process. Conversely, the TA exhibited a significant increase, likely due to the production of organic acids by lactic acid bacteria during storage, which contributed to the rise in TA and sustained a low pH environment. At day 84, the acidity of the HTHP group (6.4 g/L) was slightly lower than that of the other groups (7.0 g/L), indicating that HTHP treatment reduced the number of viable cells in the tea wine compared to other treatments, thereby decreasing the amount of organic acid produced.

### 2.2. Effects on Total Esters and Tea Polyphenols of Tea Wine

The determination of the total ester and tea polyphenol contents in tea wine is crucial for assessing its quality. Esters are the primary substances responsible for the aroma of tea wine [13]. As illustrated in Figure 1C, the total ester content of the tea wine initially increased before declining. The highest value was recorded in the CK group on the 42nd day of the storage period (5.16 g/L), while the other three groups reached their peak on the 56th day, with the LTPS group demonstrating the highest total ester content (7.27 g/L). These results indicate that the plasma-treated tea wine exhibited a greater capacity to produce aroma-presenting substances than the untreated tea wine during the storage period, resulting in an extension of the aroma presentation time by 14 days. Tea polyphenols are a significant source of antioxidants in tea wine, possessing antibacterial and detoxifying properties [14]. The diverse range of oxidized polymers that can be formed by the catechins present in tea polyphenols exert a profound influence on the color, aroma, and flavor of tea wine [15]. As shown in Figure 1D, the tea polyphenol content in tea wine exhibits a marked increase over time during storage. On day 84 of the storage period, the CK, UV, LTPS, and HTHP groups exhibited the highest values, in that order. The precipitation of tea polyphenols from tea wine during storage may be influenced by microbiological activities, such as those of *L. plantarum* and *P. pastoris*. Additionally, the fermentation conditions, bacterial strain, and initial concentration of tea polyphenols may also play a role in this process.

### 2.3. Effects on Viable Cell Counts and Alcohol Content of Tea Wine

**Figure 2:**
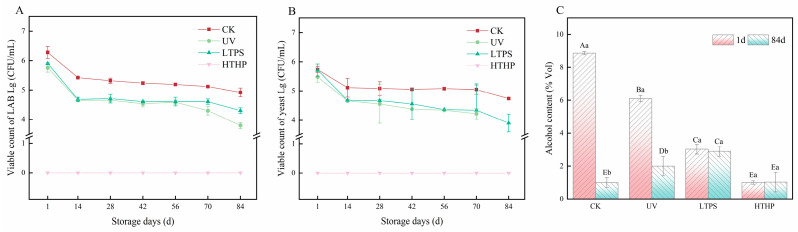
Changes in the viable cell counts and alcohol content of tea wine during storage: viable count of LAB (**A**), viable count of yeast (**B**), alcohol content (**C**). HTHP refers to high-temperature and high-pressure treatment at 121 °C for 20 min, UV refers to ultraviolet treatment for 20 min, LTPS refers to low-temperature plasma treatment at a voltage of 160 kV and a frequency of 70 Hz for 5 min, and CK refers to an untreated control. Different uppercase letters indicate group differences, different lowercase letters indicate between-group differences.

*P. pastoris* (PP), a typical non-Saccharomyces yeast, converts reducing sugars to ethanol and other metabolic by-products during alcohol fermentation, making it an excellent choice for producing complex flavors and low-alcohol wines [16]. *L. plantarum* (LP) is responsible for malolactic fermentation, which enhances microbial stability and improves the flavor profile of tea wine [17]. Therefore, determining the quantitative changes in PP and LAB in tea wine during storage is crucial for assessing its quality. As illustrated in Figure 2A,B, the populations of PP and LP in the UV, LTPS, and CK groups exhibited a gradual decline over extended storage periods. By day 84, all viable cell counts had surpassed 4 lg CFU/mL. However, the HTHP group showed undetectable LAB and yeast counts at this storage duration. These results demonstrate that LTPS and UV light treatment exhibit minimal impact on the number of viable bacteria in tea wine compared to the untreated tea wine control. Figure 2C illustrates the alcohol content of the four tea wine samples, indicating that the LTPS group was capable of maintaining a consistent alcohol content in the tea wine over 84 days, whereas the other groups exhibited a notable decline in this parameter. In particular, the alcohol content of the CK group decreased from 8.9%Vol to 1.0%Vol. It can be hypothesized that the esterification of alcohols and acids, which occurs during the storage of tea and wine, results in the production of corresponding esters. This process ultimately leads to the consumption of alcohols [18]. The alcohol content of the tea wine samples met the standards required for commercially available alcoholic beverages.

### 2.4. Effects on Color Parameters of Tea Wine

Color is one of the most important sensory attributes, often being the first feature perceived by consumers [19]. The chromaticity, hue, and lightness of tea wine can be represented by color parameters. The L*, a*, b*, and ΔE values reflect brightness, red-green hue, yellow-blue hue, and comprehensive color difference, respectively [20]. As demonstrated in Table 1, statistically significant differences were observed in the color parameters of the four groups of tea wines during the storage period (*p* < 0.05). The L* values of all tea wine samples remained within the range of 26.81 to 34.60 throughout the storage period, while the a* values ranged from −0.97 to 2.29 and the b* values ranged from 0.56 to 4.50. These findings indicate that the tea wines were notably bright and light in color, exhibiting a tendency towards a reddish-yellow hue. Furthermore, the ΔE of the LTPS group was the lowest among the four groups, suggesting that the color of the low-temperature plasma-treated tea wine remained largely unchanged during storage and was stable. This is consistent with the study by Elisa et al., where plasma treatment provided greater colour intensity [11].

### 2.5. Volatile Substances in Tea Wine

**Figure 3:**
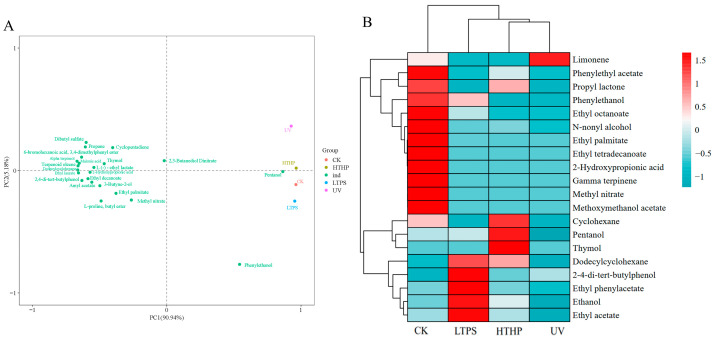
Analysis of volatile compounds identified in different tea wines: principal component (PC) biplot of the relative contents of selected major volatile compounds in different tea wine (**A**), heat map and clustering of the selected major 20 volatile compounds in tea wine (**B**). HTHP refers to high-temperature and high-pressure treatment at 121 °C for 20 min, UV refers to ultraviolet treatment for 20 min, LTPS refers to low-temperature plasma treatment at a voltage of 160 kV and a frequency of 70 Hz for 5 min, and CK refers to an untreated control.

After 84 days in storage, the differences in volatile compounds among the four tea wine samples were analyzed using HS-SPME-GC-MS (Figure 3 and Table 2), resulting in the identification of a total of 62 volatile substances categorized by their chemical structures as esters (27), olefins (18), ketones (3), alcohols (9), acids (6), and phenolics (5). It is evident that the predominant flavoring substances in tea wine are alcohols, esters, and olefins, collectively representing over 80% of the total content (Table 2). These findings align with those of Xu et al. regarding the volatile flavoring substances in black tea wine [5].

The composition and content of volatile substances in tea wine are subject to variation depending on the specific treatment employed. The observed differences among the volatile substances from the various samples underwent further analysis using principal component analysis (PCA) downscaling [21]. The results of the dimensionality reduction analysis indicated that the first two principal components explained up to 96.12% of the total variance, with PC1 accounting for 90.94% and PC2 for 5.18% (Figure 3A). The LTPS, UV, HTHP, and CK groups were clearly distinguished from one another by PC2 (5.18% of variance). The flavor score of the LTPS and CK groups demonstrated a positive correlation with 11 key volatile compounds (ethyl laurate, 2,4-di-tert-butylphenol, amyl acetate, 2-hydroxypropionic acid, ethyl decanoate, 3-butyne-2-ol, ethyl palmitate, L-proline butyl ester, methyl nitrate, pentanol, phenylethanol). Conversely, the flavor score of the UV and HTHP groups exhibited a positive correlation with the remaining nine key volatile compounds. A heat map illustrated variations in each volatile compound present across all four samples of tea wine, while cluster analysis revealed a correlation among all samples (Figure 3B), consistent with the PCA results.

Esters are key substances in the production of aroma in tea wine and are typically formed through the esterification of carboxylic acids and alcohols [18]. A total of 27 esters were identified in the four groups of tea wine samples. The esters with the highest contents were methyl nitrate, l-proline butyl ester, ethyl acetate, ethyl octanoate, L-(-)-ethyl lactate, ethyl palmitate, and so on (Table 2). Interestingly, the relative content of esters in the LTPS group (30.63%) was significantly higher than those of the other three groups (14.56%–23.16%) (Table 2). This finding aligns with the previous determinations of total esters. Among these, ethyl acetate is present in the highest relative content (24.88%), imparting a distinctive fruity flavor to the tea wine [22]. This phenomenon may be attributed to the production of a variety of reactive nitrogen species (RNS) and reactive oxygen species (ROS) by low-temperature plasma, which induces *L. plantarum* and *P. pastoris* to produce more esters [6]. Consequently, this results in a tea wine with a more pronounced flavor profile even after 84 days of storage. A comparable outcome was observed in orange juice, where dielectric barrier discharge air cold plasma treatment also preserved the aroma of the juice and extended its shelf life to 26 days [23].

Alcohols are ubiquitous products of the fermentation process of tea wine, arising from the activity of yeast and lactic acid bacteria [24]. Nine alcohol compounds, including ethanol, pentanol, phenylethanol, n-nonyl alcohol, octanol, 3-butyne-2-ol, 2,3-butanediol, 3-methylacetic acid 1-butanol, and alpha terpineol were identified in the tea wine, with the most prominent being ethanol, phenylethanol, and pentanol (Table 2). It is noteworthy that the LTPS group exhibited the highest relative ethanol content (46.22%) (Table 2), indicating that the low-temperature plasma treatment effectively maintained the alcohol content and product quality during the storage of tea wine. The production of phenylethanol and pentanol results from the degradation of phenylalanine and leucine, respectively. These compounds impart a distinctive rosy, fruity, and sweet flavor to tea wine [25].

Furthermore, only six acids were identified in the samples, and their concentrations were relatively low (Table 2). This may be attributed to the fact that the majority of acids produced by the metabolism of lactic acid bacteria in tea wine undergo reactions with alcohols to form esters [26]. Similarly, the content of olefins and phenolics in tea wine is minimal (Table 2).

Overall, it can be posited that low-temperature plasma treatment of tea wines may prove to be an effective method for retaining alcohols and esters during storage, in comparison to untreated, UV-treated, and HTHP-treated tea wines. Furthermore, it was observed that this process enhanced the fruity and sweet flavors of the tea wine. Nevertheless, further investigation is required to elucidate the detailed molecular mechanism underlying the enhancement of tea wine flavor.

### 2.6. Correlation Analysis

**Figure 4:**
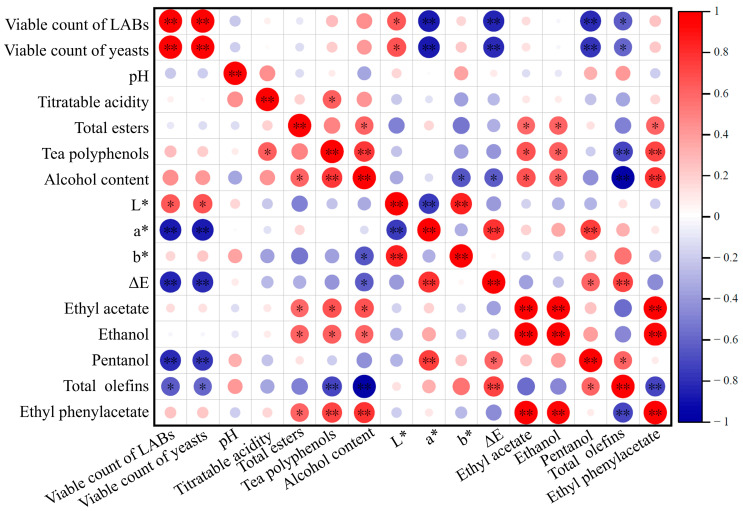
Correlation analysis of physicochemical indexes and volatile substances of tea wines treated in different ways. * *p ≤* 0.05, ** *p* ≤ 0.01.

The results of the Spearman correlation analysis are presented in Figure 4, where red (+1) indicates a strong positive correlation, blue (−1) indicates a strong negative correlation, and white (0) indicates no correlation. Ethyl phenylacetate was positively correlated with total esters, tea polyphenols, alcohol content, ethyl acetate, and ethanol and was negatively correlated with total olefins. Total olefins were positively correlated with ΔE and pentanol and were negatively correlated with the viable count of LABs, viable count of yeasts, tea polyphenols, and alcohol content. Pentanol was positively correlated with a* and ΔE and was negatively correlated with the viable count of LABs and viable count of yeasts. Ethanol and ethyl acetate were positively correlated with total esters, tea polyphenols and alcohol content. ΔE was negatively correlated with the viable count of LABs, viable count of yeasts, and alcohol content. The a* and b* were the same as ΔE; however L* was the opposite of ΔE. Alcohol content was positively correlated with total esters and tea polyphenols. Tea polyphenols were positively correlated with total esters and titratable acidity. The viable count of LABs was positively correlated with the viable count of yeasts.

## 3. Materials and Methods

### 3.1. Bacterial Strains and Growth Conditions

*Lactiplantibacillus plantarum* HYY-S10 (LP) (CGMCC No. 62784) was previously isolated from De’ang sour tea, while *Pichia pastoris* Mbpk-21 (PP) (GDMCC No.62253) was isolated from mulberry fruit wine. Both strains were stored in the Guangdong Microbial Culture Collection Center (GDMCC, Guangzhou, China) and were freeze-dried and used to prepare tea wine starters, respectively. They were activated twice and washed, then resuspend in sterile saline and set aside at 4 °C.

### 3.2. Preparation of Tea Wine

**Figure 5:**
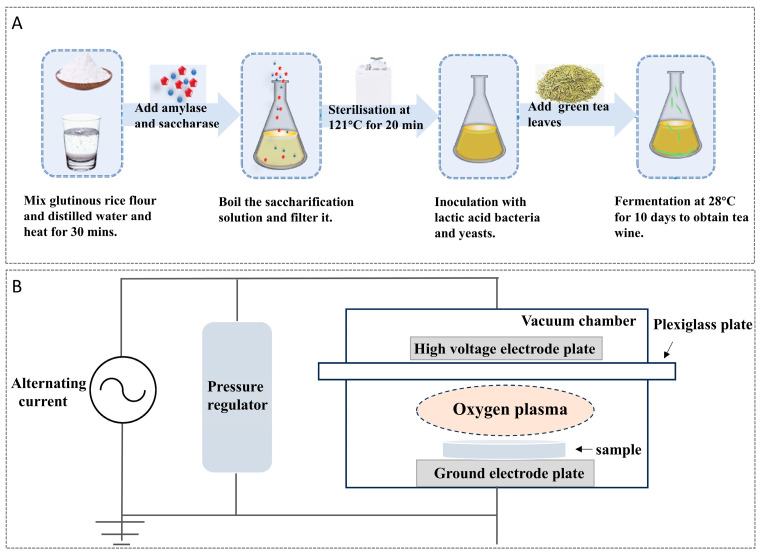
Flow chart of the preparation process of tea wine (**A**), schematic diagram of low-temperature plasma device (**B**).

The preparation of tea wine was conducted following the methodology outlined by Chen et al., with subsequent enhancements made to the process [27]. The production methodology of tea wine is illustrated in Figure 5A. A solution of glutinous rice flour and water was prepared in a 1:8 ratio (25 g:200 mL), and the mixture was subjected to a water bath at 95 °C for 30 min. Subsequently, 3.28 amylase (1000 U/g, 12.8%, glutinous rice) was added and treated with a water bath at 80 °C for 45 min. Following cooling to room temperature, 0.04 g of glucoamylase (1000 U/g, 0.16%, glutinous rice) was added and treated with a water bath at 60 °C for 210 min. Subsequently, a reaction with an iodine solution was carried out at 95 °C for 30 min. During this period, the solution was stirred continuously until no blue color was observed upon reaction with the iodine solution. The saccharification solution was boiled for a period of 10 min, filtered through four layers of 100-mesh gauze, and subsequently transferred into conical flasks for sterilization at 121 °C for a duration of 20 min. In a sterile environment, tea (2% of the feed solution) and *L. plantarum* HYY-S10 (6 lg CFU/mL) and *P. pastoris* Mbpk-21 (6 lg CFU/mL) were added. After fermentation at 28 °C for 14 days, a sample of tea wine was obtained through preliminary filtration. The tea wine samples underwent four distinct treatments: high-temperature and high-pressure (HTHP) treatment at 121 °C for 20 min, ultraviolet (UV) treatment for 20 min, low-temperature plasma (LTPS) treatment at a voltage of 160 kV and a frequency of 70 Hz for 5 min, and no treatment (CK). A schematic representation of the plasma device is presented in Figure 5B, where the LTPS processing parameters are referenced and modified from the original work of Iwona et al. [9].

All samples were stored at room temperature (25 °C) in a dry environment, protected from light, and closed for a period of 84 days. During this period, the pH, titratable acidity, total esters, tea polyphenol content, viable cell counts, alcohol content, and color parameters of the samples were determined periodically (at 1, 14, 28, 42, 56, 70, and 84 days). On the final day, samples were analyzed for volatile compounds.

### 3.3. Determination of Physicochemical Properties

#### 3.3.1. Determination of pH and Titratable Acidity

The pH of the tea wine samples was measured using a portable pH meter (RHSJ-3F, China). The titratable acidity (TA) was quantified by potentiometric titration in accordance with the methodology proposed by Huang et al. [28] and was expressed as lactic acid content (g/L). Specifically, 1 mL of the sample solution was combined with 2 mL of distilled water and two drops of phenolphthalein in a beaker and titrated with 0.01 M NaOH until the solution exhibited a slight red hue for 30 s without fading, indicating that the endpoint had been reached. A control test was performed concurrently. The TA was calculated from the consumption of NaOH using the following equation:

$$TA=V_{1}\times \frac{c}{0.1}\times \frac{100}{V_{2}}$$

where *V*_1_ represents the volume of NaOH consumed, *V*_2_ is the aspirated volume of the sample, and *c* is the concentration of NaOH (0.01 M)

#### 3.3.2. Determination of Total Esters and Tea Polyphenols

Total esters were measured using automated potentiometric titration, as described by Ouyang et al. [29], and expressed as ethyl acetate content (g/L). The tea polyphenols in tea wine were quantified following the methodology outlined by David et al. [30]. Specifically, the content of tea polyphenols, measured in mg/kg of gallic acid (TP), was determined using the Folin–Ciocalteau method with a Miura-One enzymatic autoanalyzer (BioTek Instruments, Winooski, VT, USA).

#### 3.3.3. Determination of Viable Cell Counts

Each tea wine sample (100 μL) was diluted in a sterile saline solution (0.9%). To assess the presence of lactic acid bacteria (LAB) colonies, an MRS agar plate (Guangdong Huankai Biotechnology Co., Guangzhou, China) was incubated at 37 °C for 24 h. The number of yeast colonies was quantified by incubating a Rose Bengal agar plate (Guangdong Huankai Biotechnology Co., Guangzhou, China) at 30 °C for 3 to 5 days.

#### 3.3.4. Determination of Color Parameters

After dispensing the tea wine samples into a cuvette, color measurements were taken using a colorimeter (CoLor i7, U.S. X-Rite Co., Michigan, USA) set with a measuring aperture of 6 mm and a lens of 6 mm, and three measurements were recorded.

#### 3.3.5. Determination of Alcohol Content

On the 1st day and the 84th day, tea wine samples of 100 mL were obtained for the purpose of determining the alcohol content. Non-volatile substances in the samples were removed using a rotary evaporator (RE-2000B, Shanghai Yarong Biochemical Instrument Factory, Shanghai, China). At a standard temperature (typically 20 °C), the alcohol meter (DA-130N, Kyoto Electronics Manufacturing Co., Kyoto, Japan) should be inserted vertically into the tea wine. The reading displayed after the level has reached a stable point is the alcohol content (% vol).

### 3.4. HS-SPME-GC-MS Measurement of Volatile Substances

Volatile components were determined with reference to Xu et al. [3]. Volatile substances in the four tea wine samples (CK, UV, LTPS, and HTHP groups) were measured using headspace solid-phase micro-extraction (HS-SPME) coupled with a gas chromatograph–mass spectrometer (8890B/5799B, Agilent Technologies, Santa Clara, CA, USA). The spectrometer was equipped with a ZB-WAX capillary column (30 m × 0.25 mm I.D., 0.25 μm df; Agilent Technologies) and the SPME device was equipped with fibers (5610–5874, Agilent Technologies) from Supelco Inc., Bellefonte, PA, USA.

For the analysis, each of the four tea wine samples (5.00 mL) was placed in a 20 mL headspace sample bottle and incubated at 80 °C for 40 min to facilitate the absorption of volatiles by the SPME fiber prior to GC-MS analysis. The GC conditions were established as follows: the column chamber temperature was initially set at 50 °C (held for 5 min), then increased to 220 °C at a rate of 5 °C/min (held for 15 min); the vaporization chamber temperature was set at 250 °C; the carrier gas was high purity He (99.999%) with a flow rate of 1.0 mL/min; and the injection was performed without splitting the sample. The identification of volatile substances was achieved by comparing their mass spectra, obtained through chemical ionization with methane gas. Additionally, the spectra were compared with those of authentic standards, while also considering retention times and alignment with the spectral fragmentation patterns found the NIST library database.

### 3.5. Data Analysis

The experiment was conducted in a randomized design, with each treatment being replicated three times. The data were analyzed using the statistical software package SPSS 27.0 (SPSS Inc., Chicago, IL, USA), with one-way analysis of variance employed as the analytical technique. A Duncan multiple range test was employed to ascertain whether the results of the various treatments exhibited statistically significant differences.

## 4. Conclusions

In this study, we compared the effects of different treatments on the storage period of tea wine. The application of low-temperature plasma treatment has been demonstrated to effectively maintain viable counts, color, and alcohol content during storage, while also enhancing the polyphenol content of the tea wine. The analysis of volatile compounds revealed that the relative content of alcohols and esters in the LTPS group was the highest (89.72%) during storage, thereby enhancing the fruity and sweet aroma of the tea wine. The present study offers a theoretical foundation for the utilization of low-temperature plasma technology in the context of tea wine storage. Nevertheless, further research and investigation are required to elucidate the mechanisms of low-temperature plasma action and its industrial applications.

## Figures and Tables

**Table 1:** Color changes in tea wine during storage.

Color Parameters	Storage Period (d)						
	0	14	28	42	56	70	84
CK							
L*	27.27 ± 0.01 ^a^	27.28 ± 0.06 ^a^	27.34 ± 0.00 ^a^	32.03 ± 0.04 ^c^	30.13 ± 0.03 ^b^	34.60 ± 0.03 ^d^	34.25 ± 0.07 ^d^
a*	−0.08 ± 0.04 ^a^	−0.97 ± 0.00 ^a^	−0.97 ± 0.04 ^a^	−0.40 ± 0.00 ^a^	0.30 ± 0.01 ^a^	−0.72 ± 0.00 ^a^	−0.07 ± 0.06 ^a^
b*	4.50 ± 0.00 ^c^	5.13 ± 0.03 ^d^	3.37 ± 0.00 ^b^	3.09 ± 0.03 ^b^	3.11 ± 0.03 ^b^	2.28 ± 0.04 ^a^	3.74 ± 0.03 ^b^
ΔE	-	1.17 ± 0.04 ^a^	1.76 ± 0.03 ^a^	3.74 ± 0.06 ^b^	1.14 ± 0.03 ^a^	4.65 ± 0.01 ^c^	1.63 ± 0. 01 ^a^
UV							
L*	28.92 ± 0. 01 ^c^	27.71 ± 0.03 ^b^	27.95 ± 0.05 ^b^	27.28 ± 0.00 ^b^	27.58 ± 0.03 ^b^	25.28 ± 0.05 ^a^	28.50 ± 0.06 ^c^
a*	0.29 ± 0.03 ^a^	0.16 ± 0.05 ^a^	0.23 ± 0.03 ^a^	0.18 ± 0. 01 ^a^	0.30 ± 0.03 ^a^	0.21 ± 0.06 ^a^	0.02 ± 0.07 ^a^
b*	3.37 ± 0.08 ^c^	3.62 ± 0.00 ^c^	3.61 ± 0.00 ^c^	1.67 ± 0.03 ^b^	1.90 ± 0.06 ^b^	−0.05 ± 0.03 ^a^	0.12 ± 0.03 ^a^
ΔE	-	1.24 ± 0.06 ^b^	0.25 ± 0.03 ^a^	2.05 ± 0.05 ^c^	0.39 ± 0.09 ^a^	3.01 ± 0.04 ^d^	1.69 ± 0. 01 ^b^
LTPS							
L*	28.93 ± 0.03 ^b^	27.18 ± 0.03 ^a^	28.22 ± 0.00 ^b^	27.72 ± 0.00 ^a^	27.52 ± 0.00 ^a^	26.65 ± 0.03 ^a^	28.00 ± 0.07 ^b^
a*	0.19 ± 0.00 ^a^	0.13 ± 0.01 ^a^	0.18 ± 0.03 ^a^	0.20 ± 0.03 ^a^	0.28 ± 0.08 ^a^	0.15 ± 0.05 ^a^	0.15 ± 0.03 ^a^
b*	3.50 ± 0.09 ^c^	3.65 ± 0.03 ^c^	3.54 ± 0.01 ^c^	1.93 ± 0.06 ^b^	1.81 ± 0.01 ^b^	0.63 ± 0.03 ^a^	0.56 ± 0.05 ^a^
ΔE	-	1.75 ± 0.09 ^b^	1.04 ± 0.06 ^b^	1.69 ± 0.03 ^b^	0.24 ± 0.03 ^a^	1.47 ± 0.08 ^b^	1.35 ± 0.07 ^b^
HTHP							
L*	27.94 ± 0.01 ^a^	27.33 ± 0.03 ^a^	28.47 ± 0.03 ^b^	27.55 ± 0.03 ^a^	27.73 ± 0.07 ^a^	27.34 ± 0.03 ^a^	26.81 ± 0.05 ^a^
a*	1.13 ± 0.03 ^a^	1.09 ± 0.06 ^a^	1.11 ± 0.07 ^a^	0.77 ± 0.03 ^a^	2.29 ± 0.03 ^b^	0.65 ± 0.03 ^a^	0.32 ± 0.08 ^a^
b*	3.96 ± 0.05 ^a^	3.14 ± 0.07 ^a^	3.44 ± 0.08 ^a^	2.71 ± 0.08 ^a^	3.95 ± 0.04 ^a^	1.19 ± 0.04 ^a^	1.48 ± 0.04 ^a^
ΔE	-	1.02 ± 0.05 ^a^	1.22 ± 0.03 ^a^	1.22 ± 0.07 ^a^	1.96 ± 0.03 ^a^	3.23 ± 0.08 ^a^	2.16 ± 0.03 ^a^

HTHP refers to high-temperature and high-pressure treatment at 121 °C for 20 min, UV refers to ultraviolet treatment for 20 min, LTPS refers to low-temperature plasma treatment at a voltage of 160 kV and a frequency of 70 Hz for 5 min, and CK refers to an untreated control. Different lowercase letters indicate significant differences in each row.

**Table 2:** Types and contents of the main volatile substances in tea wine.

Number	Compounds	Relative Content (%)			
		CK	LTPS	UV	HTHP
Esters					
1	Methyl nitrate	5.71	ND	ND	ND
2	L-proline, butyl ester	ND	3.78	ND	ND
3	Ethyl acetate	7.67	24.88	ND	8.49
4	Methoxymethanol acetate	0.77	ND	ND	ND
5	Ethyl octanoate	0.19	0.05	ND	ND
6	Ethyl phenylacetate	0.03	0.20	ND	0.03
7	Phenylethyl acetate	0.12	ND	ND	0.04
8	Propyl lactone	0.07	ND	ND	0.05
9	Ethyl decanoate	1.41	ND	ND	ND
10	Ethyl laurate	1.17	ND	ND	ND
11	Ethyl undecanoate	0.47	ND	ND	ND
12	Ethyl tetradecanoate	0.63	ND	ND	ND
13	9-Hexadecaenoic acid ethyl ester	0.21	ND	ND	ND
14	Ethyl palmitate	4.13	0.07	ND	ND
15	N (2,5-difluoromethylbenzoyl)-amyl ester	0.58	ND	ND	ND
16	L (-) -ethyl lactate	ND	0.43	ND	1.79
17	Amyl acetate	ND	1.09	ND	ND
18	Linalyl formate	ND	0.07	ND	ND
19	Dodecyl pentyl sulfite	ND	0.06	ND	ND
20	ethylidene diacetate	ND	ND	11.44	ND
21	Diallyl oxalate	ND	ND	0.48	ND
22	Dibutyl sulfate	ND	ND	1.13	ND
23	Ethyl isocyanate	ND	ND	0.59	ND
24	Propyl lactate	ND	ND	0.44	ND
25	6-bromohexanoic acid, 3,4-dimethylphenyl ester	ND	ND	0.50	0.23
26	2,3-Butanediol Dinitrate	ND	ND	ND	9.67
27	Diethyl succinate	ND	ND	ND	0.02
Total esters		23.16	30.63	14.58	20.32
Olefins					
28	3-chloropropyne	0.25	ND	ND	ND
29	2-Propene-1-ol	1.23	ND	ND	ND
30	3,4-(1,1-dimethylethyl)-2,2,5,5-tetramethylhexane	0.78	ND	ND	ND
31	1,2-dimethylepoxyethane	0.22	ND	ND	ND
32	1-butene	ND	ND	ND	0.28
33	Cyclopentadimethylsiloxane	0.06	ND	ND	ND
34	Gamma terpinene	0.19	ND	ND	ND
35	3-carene	0.16	ND	ND	ND
36	Dodecylcyclohexane	0.04	0.4	ND	0.31
37	Limonene	0.04	ND	0.08	ND
38	Cyclohexane	0.16	ND	ND	0.25
39	Dodecane	0.03	0.09	ND	ND
40	1-isopropyl-2,3-dimethylcyclopentane	0.19	ND	ND	ND
41	4-methyl-2-dodecene	ND	0.13	ND	ND
42	Terpenoid oleene	ND	0.17	ND	0.37
43	3-Chloro-4,4-dimethoxy-2-methyl-1-butene	ND	ND	0.14	ND
44	Cyclopentadiene	ND	ND	1.02	2.97
45	Propane	ND	ND	0.81	0.28
Total olefins		3.58	0.65	2.10	4.56
Acids					
46	2-Hydroxypropionic acid	1.12	ND	ND	ND
47	Thiosalicylic acid	0.02	ND	ND	ND
48	Ethyl boronic acid	ND	0.17	ND	ND
49	2-ethylheptanoic acid	ND	0.05	ND	ND
50	1,1,2-Trimethylhydrazine hydrochloride	ND	ND	0.12	ND
51	Malonic acid	ND	ND	0.25	0.03
Total acids		1.14	0.23	0.37	0.03
Phenolics					
52	2,4-di-tert-butylphenol	ND	1.34	0.40	0.23
53	Thymol	ND	ND	ND	3.23
Total phenolics		ND	1.34	0.40	3.46
Alcohols					
54	Ethanol	35.56	46.22	32.21	38.46
55	Pentanol	5.41	5.57	3.40	8.42
56	Phenylethanol	11.19	7.18	0.19	ND
57	N-nonyl alcohol	0.65	0.06	ND	0.09
58	Octanol	0.28	0.06	ND	0.04
59	3-Butyne-2-ol	2.57	ND	ND	ND
60	2,3-Butanediol	ND	ND	0.11	ND
61	3-Methylacetic acid 1-butanol	ND	ND	0.17	ND
62	Alpha terpineol	ND	ND	0.11	0.42
Total alcohols		55.66	59.09	36.19	47.43

“ND”: not detected. HTHP refers to high-temperature and high-pressure treatment at 121 °C for 20 min, UV refers to ultraviolet treatment for 20 min, LTPS refers to low-temperature plasma treatment at a voltage of 160 kV and a frequency of 70 Hz for 5 min, and CK refers to an untreated control.

## Data Availability

Research data are not shared.

## References

[1] Chen Q., Sun C., Ouyang Q., Wang Y., Liu A., Li H., Zhao J. (2015). Classification of different varieties of Oolong tea using novel artificial sensing tools and data fusion. LWT Food Sci. Technol..

[2] Yang Y., Ye Z., Qin Y., Pathirana S., Araujo L.D., Culley N.J., Kilmartin P.A. (2024). Effects of post-fermentation addition of green tea extract for sulfur dioxide replacement on Sauvignon Blanc wine phenolic composition, antioxidant capacity, color, and mouthfeel attributes. Food Chem..

[3] Xu W., Wang X., Jia W., Wen B., Liao S., Zhao Y., Tang Q., Li K., Hua Y., Yang Y. (2023). Dynamic changes in the major chemical and volatile components during the “Ziyan” tea wine processing. LWT Food Sci. Technol..

[4] Xu X., Ma W.L., Hu S.N., Qian Z.Y., Shen C., Mao J. (2021). Neuroprotective effects of Chinese rice wine fermented with Fu Brick tea on H_2_O_2_-induced PC12 Cells. FASEB J..

[5] Xu W., Zhu Y., Lin L., Tunyaluk B., Li P. (2024). Dynamic changes in volatile components during dark tea wine processing. LWT Food Sci. Technol..

[6] Shabbir A., Hassan S.A., Hanif H., Rauf R., Muntaha S.T., Jubbar M., Aadil R.M. (2024). Applications of cold plasma technique to enhance the safety and quality of different food products. Meas. Foods.

[7] He F., Liang N.-N., Mu L., Pan Q.-H., Wang J., Reeves M.J., Duan C.-Q. (2012). Anthocyanins and their variation in red wines I. monomeric anthocyanins and their color expression. Molecules.

[8] Bao Y., Reddivari L., Huang J.-Y. (2020). Enhancement of phenolic compounds extraction from grape pomace by high voltage atmospheric cold plasma. LWT Food Sci. Technol..

[9] Niedźwiedź I., Płotka-Wasylka J., Kapusta I., Simeonov V., Stój A., Waśko A., Pawłat J., Polak-Berecka M. (2022). The impact of cold plasma on the phenolic composition and biogenic amine content of red wine. Food Chem..

[10] Lukić K., Vukušić T., Tomašević M., Ćurko N., Gracin L., Kovačević Ganić K. (2019). The impact of high voltage electrical discharge plasma on the chromatic characteristics and phenolic composition of red and white wines. Innov. Food Sci. Emerg..

[11] Sainz-García E., López-Alfaro I., Múgica-Vidal R., López R., Escribano-Viana R., Portu J., Alba-Elías F., González-Arenzana L. (2019). Effect of the atmospheric pressure cold plasma treatment on Tempranillo red wine quality in batch and flow systems. Beverages.

[12] Luo W., Wu W., Du X., Yu Y., Wu J., Xu Y., Li L. (2023). Regulation of the nitrite, biogenic amine and flavor quality of Cantonese pickle by selected lactic acid bacteria. Food Biosci..

[13] Qu J., Chen X., Wang X., He S., Tao Y., Jin G. (2024). Esters and higher alcohols regulation to enhance wine fruity aroma based on oxidation-reduction potential. LWT Food Sci. Technol..

[14] Aloo O.S., Kim D.-G., Vijayalakshmi S., Aloo D.O., Ochola C.O., Oh D.-H. (2024). Polyphenol constituents and impacts of fermented teas (*Camellia sinensis*) in human wellness. Food Biosci..

[15] Jiang H., Xu W., Chen Q. (2020). Determination of tea polyphenols in green tea by homemade color sensitive sensor combined with multivariate analysis. Food Chem..

[16] Wang X., Fan G., Peng Y., Xu N., Xie Y., Zhou H., Liang H., Zhan J., Huang W., You Y. (2023). Mechanisms and effects of non-*Saccharomyces* yeast fermentation on the aromatic profile of wine. J. Food Compos. Anal..

[17] Chen L., Li K., Chen H., Li Z. (2023). Reviewing the source, physiological characteristics, and aroma production mechanisms of aroma-producing yeasts. Foods.

[18] Zhang B., Zheng S., Huang M., Wu Q., Dong W., Wu J., Liu H., Zhao D., Yu Y., Li J. (2024). Analysis of volatile compounds in Xiangjiao baijiu from different storage containers and years based on HS-GC-IMS and DI-GC-MS. Food Chem. X.

[19] Parpinello G.P., Versari A., Chinnici F., Galassi S. (2009). Relationship among sensory descriptors, consumer preference and color parameters of Italian Novello red wines. Food Res. Int..

[20] Xia N.-Y., Liu A.-Y., Qi M.-Y., Zhang H.-L., Huang Y.-C., He F., Duan C.-Q., Pan Q.-H. (2024). Enhancing the color and astringency of red wines through white grape seeds addition: Repurposing wine production byproducts. Food Chem. X.

[21] Huang Y.-Y., Jia X.-Z., Yu J.-J., Chen Y.-H., Liu D.-M., Liang M.-H. (2021). Effect of different lactic acid bacteria on nitrite degradation, volatile profiles, and sensory quality in Chinese traditional paocai. LWT Food Sci. Technol..

[22] Zhang B.Q., Tang C., Yang D.Q., Liu H., Xue J., Duan C.Q., Yan G.L. (2022). Effects of three indigenous non-*Saccharomyces* yeasts and their pairwise combinations in co-fermentation with *Saccharomyces cerevisiae* on volatile compounds of Petit Manseng wines. Food Chem..

[23] Dzimitrowicz A., Pohl P., Caban M., Jamroz P., Cyganowski P., Bykowski M., Klimczak A., Bielawska-Pohl A. (2022). How does direct current atmospheric pressure glow discharge application influence on physicochemical, nutritional, microbiological, and cytotoxic properties of orange juice?. Food Chem..

[24] Jiang J., Yin R., Xie Y., Ma X., Cui M., Chen Y., Li Y., Hu Y., Niu J., Cheng W. (2024). Effects of cofermentation of *Saccharomyces cerevisiae* and different lactic acid bacteria on the organic acid content, soluble sugar content, biogenic amines, phenol content, antioxidant activity and aroma of prune wine. Food Chem. X.

[25] Wang F., Zhao P., Du G., Zhai J., Guo Y., Wang X. (2024). Advancements and challenges for brewing aroma-enhancement fruit wines: Microbial metabolizing and brewing techniques. Food Chem..

[26] Huang Y.-Y., Yu J.-J., Zhou Q.-Y., Sun L.-N., Liu D.-M., Liang M.-H. (2020). Preparation of yogurt-flavored bases by mixed lactic acid bacteria with the addition of lipase. LWT Food Sci. Technol..

[27] Chen L., Li D., Ren L., Song S., Ma X., Rong Y. (2021). Effects of simultaneous and sequential cofermentation of *Wickerhamomyces anomalus* and *Saccharomyces cerevisiae* on physicochemical and flavor properties of rice wine. Food Sci. Nutr..

[28] Huang Y.-Y., Huang F., Huang J.-Y., Liu Y., Li M.-N., Yao Q.-B., Zeng X.-A., Chen B.-R. (2024). Repercussions of *Lactiplantibacillus plantarum* HYY-DB9 and *Levilactobacillus brevis* GIM 1.773 on the degradation of nitrite, volatile compounds, and sensory assessment of traditional Chinese paocai. Int. J. Food Sci. Technol..

[29] Ouyang X., Zhu B., Liu R., Gao Q., Lin G., Wu J., Hu Z., Zhang B. (2018). Comparison of volatile composition and color attributes of mulberry wine fermented by different commercial yeasts. J. Food Process. Preserv..

[30] David I.G., Bizgan A.-M.C., Popa D.E., Buleandra M., Moldovan Z., Badea I.A., Tekiner T.A., Basaga H., Ciucu A.A. (2015). Rapid determination of total polyphenolic content in tea samples based on caffeic acid voltammetric behaviour on a disposable graphite electrode. Food Chem..

